# The small dense LDL particle/large buoyant LDL particle ratio is associated with glucose metabolic status in pregnancy

**DOI:** 10.1186/s12944-017-0627-y

**Published:** 2017-12-14

**Authors:** Yanmin Chen, Mengkai Du, Jianyun Xu, Danqing Chen

**Affiliations:** Obstetrical Department, Women’s Hospital, School of Medicine, Zhejiang University, Hangzhou, Zhejiang Province 310006 China

**Keywords:** Lipoprotein particle, Gestational diabetes mellitus, Pregnancy, Insulin resistance

## Abstract

**Background:**

The lipoprotein subfraction particle profile can be used to improve clinical assessments of cardiovascular disease risk and contribute to early detection of atherogenic dyslipidemia. Lipid alterations in gestational diabetes have been extensively studied, but the results have been inconsistent. Here, we investigated serum lipoprotein subfraction particle levels and their association with glucose metabolic status in pregnancy.

**Methods:**

Twenty-eight pregnant women with gestational diabetes and 56 pregnant women with normal glucose tolerance matched for body mass index were enrolled in this study. We assessed fasting serum lipid concentrations and lipoprotein subfraction particle levels in participants between 24 and 28 weeks of gestation.

**Results:**

The level of low-density lipoprotein (LDL) cholesterol was significantly lower in women with gestational diabetes than in those with normal glucose tolerance, but the triglyceride and high-density lipoprotein (HDL) cholesterol levels of the two groups were similar. Lipoprotein particle analysis showed that very-low-density lipoprotein (VLDL) particle number and the small dense LDL particle/large buoyant LDL particle (sdLDL-P/lbLDL-P) ratio were significantly higher in women with gestational diabetes than in those with normal glucose tolerance (*P* = 0.013 and *P* = 0.015, respectively). In multivariate analysis, fasting glucose was independently and positively associated with sdLDL-P/lbLDL-P ratio even after adjustment for maternal age, gestational weight gain, BMI and LDL cholesterol (standardized Beta = 0.214, *P* = 0.029).

**Conclusions:**

The sdLDL-P/lbLDL-P ratio is higher in GDM compared with non-diabetic pregnant women, and positively and independently associated with fasting glucose in pregnant women.

## Background

Lipoproteins are comprised of multiple subfractions according to their size, density, and physicochemical properties. Determination of lipoprotein particle subfractions improves the clinical evaluation of individuals at high risk of cardiovascular diseases (CVD) and type 2 diabetes [1, 2]. Low-density lipoprotein (LDL) cholesterol is universally known as a major risk factor for CVD and type 2 diabetes, whereas LDL particle is more valuable as a risk indicator of LDL-attributable atherosclerosis [3]. Further, the small dense LDL (sdLDL) subfraction shows a positive association with coronary artery disease and is thought to be an atherogenic lipoprotein [4, 5]. Moreover, the protective effect of high-density lipoprotein (HDL) cholesterol on the incidence of type 2 diabetes is largely attributable to high HDL2 cholesterol subfractions [6].

Gestational diabetes mellitus (GDM), which is defined as impaired glucose tolerance that first appears during pregnancy, is associated with insulin resistance and beta-cell decompensation during pregnancy. There is a substantially increased risk of postpartum diabetes and CVD in GDM patients due, in part, to aberrant metabolic disturbances during pregnancy [7, 8]. In addition to dysglycemia, multiple metabolic and inflammatory factors are altered in women with GDM. Lipid alterations in GDM compared with normal pregnancy have been extensively studied, but the results have been inconsistent, especially those concerning LDL cholesterol [9]. The characterization of changes in lipoprotein particle levels in GDM may help identify lipid metabolic changes and potentially improve predictions of the risks of adverse pregnancy outcomes and postpartum metabolic diseases. Previous data obtained in American and Mediterranean populations indicated that women with GDM show remarkable changes in LDL particle distribution [10, 11]. However, the lipoprotein particle diameter differs according to ethnicity [12], and there are no published studies that describe the lipoprotein particle profile of the Chinese Han population with GDM. Thus, the objective of this study was to investigate differences in lipoprotein particle profile of GDM patients and healthy pregnant women and to examine the relationship between lipoprotein subfraction particles and the parameters of glucose metabolism during pregnancy.

## Methods

This study was conducted at Women’s Hospital Schools of Medicine Zhejiang University. Approval from the Institutional Ethics Committee at Women’s Hospital School of Medicine Zhejiang University was obtained, and all subjects provided informed consent. Twenty-eight singleton pregnant women with GDM were included in the present study. As controls, we selected 56 singleton pregnant women with normal glucose tolerance, matched for body mass index (BMI) and gestational age. GDM was diagnosed between the 24th and 28th weeks of gestation if one or more of the blood glucose levels measured during the one-step 75-g oral glucose tolerance test (OGTT) met or exceeded the following criteria: fasting, 5.1 mmol/L; 1-h, 10.0 mmol/L; and 2-h, 8.5 mmol/L. All participants resided in the Zhejiang Province and were of Han ethnicity. We excluded patients with obesity (BMI > 30 kg/m^2^), diabetes, thyroid disease, hypertensive disorders, renal diseases, hepatitis or other serious medical condition. In addition, any participant who had smoked or received the lipid lowering drugs, diabetic drugs during pregnancy was also excluded.

Blood samples were collected from participants at 24–28 weeks of gestation after a 12-h fast. Lipoprotein component analysis was performed using the lipoprotein subgroup particle number analysis method (SpectraCell Laboratories; Houston, TX, USA) according to a patented procedure (Patent No.: US 7,856,323 B2) [13]. The serum samples were mixed with a fluorescent dye and a gradient material and then separated in a continuous gradient (*d* = 1.000–1.300 g/cm^3^) through analytical ultracentrifugation. Then, the fluorescence of the lipoprotein particles was measured in a high-performance liquid chromatography-type flow system and normalized to a cholesterol scale using a proprietary algorithm. The lipoprotein particle profile included the quantitation of the levels of very-low-density lipoprotein particle (VLDL-P), total LDL particle (LDL-P), remnant lipoprotein particle (RLP), LDL III particle (LDL_III_-P), LDL IV particle (LDL_IV_-P), total HDL particle (HDL-P), large buoyant HDL 2b particle (lbHDL_2b_-P), and non-HDL particle. Small dense LDL particle (sdLDL-P) levels were calculated as follows: sdLDL-P = LDL_III_ particle + LDL_IV_ particle. Large buoyant LDL particle (lbLDL-P) number was calculated by subtracting sdLDL-P and RLP from total LDL particle. The coefficient of variation for lipoprotein particle analysis using known standards has been reported as ranging from 2% to 3%. Serum total cholesterol, LDL cholesterol, HDL cholesterol, triglycerides, insulin, lipoprotein(a) (Lp(a)), homocysteine, and high-sensitivity C-reactive protein (hsCRP) levels were determined using an Olympus AU400e chemistry immune analyzer (Tokyo, Japan). Blood glucose was measured using an Architect c16000 automated analyzer (Abbott Laboratories, Abbott Park, IL, USA). The homeostatic model assessment of insulin resistance (HOMA-IR) index was calculated as follows: HOMA-IR = [fasting glucose (mmol/L) × fasting insulin (μIU/mL)]/22.5.

Distribution of continuous variables was tested for normality using the Shapiro–Wilk test, and data showing a normal distribution were subjected to independent-sample t-tests. When the distribution was asymmetric, the two-sample Mann–Whitney U test was performed. The chi-square test was used for categorical variables. Correlation analyses between two parameters were evaluated using the Pearson correlation tests, after square root or logarithmic transformation of variables (whenever necessary). To evaluate the contribution of different variables to sdLDL-P/lbLDL-P ratio and VLDL-P, multivariate linear regression analyses were performed, using stepwise selection. Maternal age, gestational weight gain, BMI at OGTT, progesterone treatment history, FBG and LDL cholesterol were included as independent variables in the sdLDL-P/lbLDL-P ratio regression model. Maternal age, gestational weight gain, BMI at OGTT, progesterone treatment history, FINS and 2 h–OGTT were included as independent variables in the VLDL-P regression model. Two-sided *p*-value <0.05 was considered to indicate statistical significance. All tests were performed using the SPSS statistics version 20 (SPSS Inc., Chicago, IL, USA). The post hoc analysis was performed on the correlations to assess the appropriateness of the total sample size by determining the correlation coefficient that could be detected with 75% power using two-sided comparison with alpha = 0.05 [14].

## Results

The characteristics of the study population are described in Table 1. The maternal age of the GDM group was significantly higher (*P* = 0.005), whereas the prevalence of mothers aged ≥35 years did not significantly differ between the two groups. The history of progesterone treatment in the early gestation were found in two women with gestational diabetes compared with one control subject, which had no significant difference. Both pre-pregnancy BMI and BMI at the OGTT test were similar in the two groups. Compared with normal pregnant women, patients with GDM had higher fasting insulin (FINS) (*P* = 0.033), HOMA-IR (*P* = 0.012), and glucose values at the three time points of the OGTT (all *P* ≤ 0.001). Unexpectedly, GDM patients had significantly lower LDL cholesterol compared to those with normal glucose tolerance (*P* = 0.020). In contrast, there were no significant differences in total cholesterol, HDL cholesterol, non-HDL cholesterol, or triglyceride between the two groups. Additionally, no significant differences were found in concentrations of Lp(a) or hsCRP. Homocysteine and the triglyceride/HDL cholesterol ratio were significantly higher in the GDM group (*P* = 0.049 and *P* = 0.029, respectively).

**Table 1:** Clinical and Laboratory characteristics of the GDM and normal pregnant groups

Characteristics	Control (*n* = 56)	GDM (*n* = 28)	*p* value
Age (years)	30.0 (28.0, 33.0)	33.0 (30.3, 36.0)	0.005*
Advanced maternal age (≥ 35 years), n (%)	10 (17.8%)	9 (32.1%)	0.140
Gestational age (weeks)	25 (24, 27)	25 (24, 26)	0.817
Pre-pregnancy BMI (kg/m^2^)	20.1 ± 2.2	20.6 ± 2.5	0.392
BMI at OGTT (kg/m^2^)	23.3 ± 2.5	24.0 ± 2.5	0.148
Progesterone treatment history	1 (1.79%)	2 (7.14%)	0.257
Fasting blood glucose (mmol/L)	4.39 (4.20, 4.65)	4.66 (4.46, 5.01)	0.001*
1-h blood glucose (mmol/L)	7.24 ± 1.31	10.63 ± 1.44	<0.001*
2-h blood glucose (mmol/L)	6.35 ± 0.95	8.67 ± 1.29	<0.001*
Fasting insulin (μIU/mL)	8.1 (5.2, 11.7)	10.0 (7.0, 14.1)	0.033*
HOMA-IR	1.55 (1.01, 2.35)	2.17 (1.39, 2.94)	0.012*
Total cholesterol (mg/dL)	240.59 ± 42.69	222.96 ± 36.21	0.065
LDL cholesterol (mg/dL)	115.00 ± 35.78	96.61 ± 28.65	0.020*
HDL cholesterol (mg/dL)	84.79 ± 18.96	79.43 ± 17.35	0.213
Non-HDL cholesterol (mg/dL)	155.80 ± 37.75	143.54 ± 27.69	0.131
Triglyceride (mg/dL)	185.0 (146.5, 236.0)	219.5 (175.8, 285.3)	0.055
Lipoprotein(a) (mg/dL)	6.2 (2.5, 11.6)	6.0 (2.5, 12.5)	0.906
hsCRP (mg/L)	2.1 (1.2, 4.0)	1.8 (1.1, 3.9)	0.632
Homocysteine (μmol/L)	4.0 (3.7, 4.7)	4.8 (3.7, 5.9)	0.049*
TG/HDL cholesterol ratio	2.16 (1.64, 3.10)	2.96 (2.14, 3.84)	0.029*

Data are presented as median (interquartile range) or n (%) or mean ± SD. *BMI*, body mass index; *OGTT*, oral glucose tolerance test; *HOMA-IR*, homeostatic model assessment of insulin resistance; *LDL*, low-density lipoprotein; *HDL*, high-density lipoprotein; *hsCRP*, high-sensitivity C-reactive protein. *P* values in bold indicate significant differences (*P* < 0.05)

Further analyses of lipoprotein particle numbers between two groups are presented in Table 2. As shown in Table 2, patients with GDM showed increased VLDL-P (*P* = 0.013) and decreased total LDL-P (*P* = 0.024), whereas no significant differences were found in HDL-P level. Furthermore, we studied the subfractions of LDL and HDL particle. There were no significant differences in the levels of RLP, LDL_III_-P, LDL_IV_-P or sdLDL-P between the two groups, whereas the sdLDL-P/lbLDL-P ratio was significantly higher in GDM patients (*P* = 0.015). Despite the comparable lbHDL_2b_-*P* values of the two groups, the HDL_2b_-P/HDL-P ratio was significantly lower in the GDM group compared with the group of normal pregnant women (*P* = 0.0498).

**Table 2:** Lipoprotein particle numbers of subjects included in the study

	Control (*n* = 56)	GDM (*n* = 28)	*p* value
VLDL-P (nmol/L)	76 (53, 107)	97 (72, 142)	0.013*
Total LDL-P (nmol/L)	1160 ± 227	1042 ± 207	0.024*
RLP (nmol/L)	145 (113, 199)	175 (107, 226)	0.286
lbLDL-P (nmol/L)	401 (299, 509)	282 (227, 441)	0.005*
LDL_III_-P (nmol/L)	395 (316, 467)	380 (317, 462)	0.690
LDL_IV_-P (nmol/L)	175 (150, 209)	163 (132, 184)	0.064
LDL_IV_-P/Total LDL-P ratio	0.16 (0.13, 0.18)	0.15 (0.13, 0.18)	0.690
sdLDL-P (nmol/L)	575 (509, 644)	526 (485, 629)	0.107
sdLDL-P /Total LDL-P	0.51 ± 0.08	0.53 ± 0.06	0.271
sdLDL-P /lbLDL-P	1.40 (1.14, 1.95)	1.90 (1.33, 2.55)	0.015*
Total HDL-P (nmol/L)	8062 ± 731	8250 ± 734	0.271
lbHDL_2b_-P (nmol/L)	3661 ± 592	3543 ± 596	0.391
lbHDL_2b_-P /Total HDL-P ratio	0.46 ± 0.05	0.43 ± 0.05	0.0498*
Non-HDL-P (nmol/L)	1241 ± 231	1146 ± 208	0.070

Data are presented as median (interquartile range) or mean ± SD. VLDL-P, very-low-density lipoprotein particle; RLP, remnant lipoprotein particle; lbLDL-P, large buoyant low-density lipoprotein particle; sdLDL-P, small dense low-density lipoprotein particle; lbHDL2b-P, large buoyant high-density lipoprotein particle. *P* values in bold indicate significant differences (*P* < 0.05)

The Pearson correlations between lipoprotein particle levels and glucose metabolic parameters are presented in Table 3. The number of VLDL-P was positively correlated with FINS (*r* = 0.293, *P* = 0.007), 2-h blood glucose (*r* = 0.286, *P* = 0.009) and HOMA-IR index (*r* = 0.308, *P* = 0.005) in the pooled sample. The sdLDL-P/lbLDL-P ratio was positively correlated with FBG (*r* = 0.297, *P* = 0.006), FINS (*r* = 0.236, *P* = 0.031) and HOMA-IR index (*r* = 0.270, *P* = 0.013). Multiple linear regression analysis revealed that FBG remained independently and positively associated with sdLDL-P/lbLDL-P ratio after adjusting for age, BMI, gestational weight gain, progesterone history and LDL cholesterol (Table 4). Blood glucose at 2 h of OGTT test was associated with VLDL-P number after adjusting for age, BMI, gestational weight gain, progesterone history and FINS (Table 5). The post hoc power analysis indicated that the study had a power of 75% to detect a correlation coefficient of ≥0.285 with a total sample size of 84 subjects.

**Table 3:** Pearson correlations between lipoprotein particle profile and glucose metabolic parameters

	FBG^a^	FINS^a^	2-h OGTT glucose	HOMA-IR^a^
VLDL-P (nmol/L)^b^	0.209	0.293**	0.286**	0.308**
Total LDL-P (nmol/L)	−0.136	−0.062	−0.097	−0.080
lbLDL-P (nmol/L)^b^	−0.261*	−0.197	−0.178	−0.227*
sdLDL-P /lbLDL-P ratio^b^	0.297**	0.236*	0.158	0.270**
HDL2b-P (%)	0.029	0.098	0.015	0.096

*P* values in bold indicate significant differences. *, *P* < 0.05, **, *P* < 0.01

^a^Skewed variables were logarithmically transformed before testing

^b^Skewed variables were square root transformed before testing

**Table 4:** Multivariate regression analysis with sdLDL/lbLDL ratio as dependent variable^a^

Independent variables	*β*	*P*
Age^b^	−0.062	0.544
BMI	0.068	0.496
Gestational weight gain	0.009	0.930
Progesterone history	0.003	0.971
FBG^b^	**0.214**	0.029*
LDL cholesterol	**−0.450**	<0.001***

^a^Standardized *β*-coefficients and *P* values are given

^b^Skewed variables were logarithmically transformed before testing. *, *P* < 0.05, ***, *P* < 0.001

**Table 5:** Multivariate regression analysis with VLDL particle number as dependent variable^a^

Independent variables	*β*	*P*
Age^b^	−0.018	0.875
BMI	0.058	0.600
Gestational weight gain	0.032	0.775
Progesterone history	−0.137	0.204
FINS^b^	0.201	0.077
2 h OGTT	**0.284**	0.01*

^a^Standardized *β*-coefficients and *P* values are given

^b^Skewed variables were logarithmically transformed before testing. *, *P* < 0.05

## Discussion

In the current study, we investigated lipid profiles, including conventional lipid measurements and lipoprotein particle levels, in women with GDM and in pregnant women with normal glucose tolerance. The results indicated that the VLDL-P number and sdLDL-P/lbLDL-P ratio of those with and without GDM significantly differed and were both correlated with glucose metabolic parameters.

There is evidence that maternal lipid levels become abnormal in the context of GDM. A recent meta-analysis reported that GDM patients had higher triglyceride and lower HDL cholesterol levels than pregnant women with normal glucose tolerance in the second trimester; yet, both total cholesterol and LDL cholesterol levels were inconsistent [9]. Our results showed a significant decrease in LDL cholesterol with GDM, which was counterintuitive but in agreement with some previous studies [10, 15, 16]. Moreover, White et al. [17] previously reported that increased LDL cholesterol levels were associated with a lower risk of type 2 diabetes. Several genetic variants that have been associated with reduced LDL cholesterol have also been associated with increased risk of diabetes [18]. These studies help explain the phenomenon of decreased LDL cholesterol during GDM with the increased risk of developing diabetes postpartum.

Although three studies have investigated the relationship between LDL subfractions and GDM, there is no data concerning lipoprotein particle levels in the Chinese Han population. Rizzo et al. [11] reported an increased number but decreased average size of small dense LDL particles in Mediterranean women with GDM whose triglyceride and LDL cholesterol were similar to those of normal pregnant women. In a study conducted by Qiu et al. [10], American patients with GDM showed higher triglyceride and lower LDL cholesterol as well as smaller LDL particle size compared with pregnant women with normal glucose tolerance. Additionally, Han et al. [19] found that women had a smaller LDL peak diameter size and a higher level of the small dense LDL subfraction years before developing GDM. In this study, we used a different lipoprotein particle subfraction parameter, the sdLDL-P/lbLDL-P ratio, to reflect LDL particle mean size. Although the number of total LDL particles was decreased in the GDM group, the sdLDL-P/lbLDL-P ratio was significantly increased, which indicated a tendency toward the predominance of small dense LDL particles.

Previous studies have reported that the ratio of small dense LDL to large buoyant LDL is a potent marker for evaluating lipid metabolic status. A recent study by Lee et al. [20] determined that the sdLDL/lbLDL ratio increases during the development of impaired fasting glucose and is strongly associated with insulin resistance, suggesting atherogenic dyslipidemia from a pre-diabetic stage. A high sdLDL/lbLDL ratio has also been shown to be associated with lipid metabolic disturbance in patients with metabolic syndrome and human immunodeficiency virus (HIV)-associated lipodystrophy [21, 22]. Although a different analytic methodology was used to measure the number of lipoprotein particle in this study, the sdLDL-P/lbLDL-P ratio in pregnant women was positively correlated with fasting glucose after adjusting for age and BMI, two factors known to affect lipid metabolism. It seems plausible that alteration in LDL particle size would be linked to glucose metabolic status.

The causal relationship between abnormal lipoprotein patterns and CVD has been firmly established, and the lipoprotein profile has also been well studied in type 2 diabetes. The exacerbation of lipoprotein patterns is primarily attributed to insulin resistance in type 2 diabetes. With aggravating insulin resistance, the concentration and mean size of VLDL-P increase, whereas LDL and HDL particle sizes decrease [23]. Abnormal lipoprotein profiles, including higher small dense LDL and lower large buoyant HDL fractions, indicate poor glycemic control in overweight adolescents with type 2 diabetes [24]. A recent article by Mackey et al. [25] described the association between lipoprotein particles and the incidence of type 2 diabetes in a multicenter prospective cohort study, indicating that the number and size of VLDL-P were significantly associated with the incidence of type 2 diabetes in patients with atherosclerosis. Thus, it is justifiable to speculate aberrant lipoprotein particle profile existing at the early gestation in the subjects who developing gestational diabetes. The sdLDL-P/lbLDL-P ratio and VLDL-P number may be potential as biomarkers to improve early intervention of gestational diabetes.

Nutraceuticals have been shown a peculiar role in ameliorating dyslipidemia, also in pregnant women [26]. It was recently reported that omega-3 fatty acids and vitamin E co-supplementation had beneficial effects on glucose homeostasis parameters, serum triglycerides and VLDL cholesterol in GDM women [27]. Moreover, vitamin D and symbiotic supplementation were of some use for improve lipid profile among GDM patients [28, 29]. These data suggest that nutraceuticals and dietary intervention may modulates maternal glucose and lipid metabolism, but more randomized trials are needed to evaluate the effects of nutraceuticals [30]. In our study, as the subjects received similar dietary advice at obstetric clinic, we have not assessed nutraceutical intakes or taken this into account as a confounding variable.

Our study has several limitations. Firstly, because of its cross-sectional design, we were unable to determine whether abnormal lipoprotein subfractions are a cause or a consequence of impaired glucose metabolic status. Secondly, the relatively small sample size may have limited the generalization of our current findings. However, we were able to identify the correlations between lipoprotein parameters and glucose metabolic parameters. Meanwhile, the post hoc analysis showed that the study had a power of 75% to detect a correlation coefficient with *r* ≥ 0.285, implying that statistical power should not be a serious problem. Further cohort studies with a larger sample size and participants in their three trimesters of pregnancy and postpartum period are warranted to investigate the dynamic alterations of lipoprotein subfraction profiles in the perinatal period.

## Conclusion

In conclusion, our study described the lipoprotein subfraction particle profile in pregnant women and its relationship to glucose metabolic parameters. The sdLDL-P/lbLDL-P ratio is independently and positively associated with fasting glucose in pregnant women. Our study suggests that GDM patients have a tendency toward the predominance of small dense LDL particles, which may contribute to an increased risk for atherosclerosis and CVD in the postpartum period.

## Abbreviations

BMI: Body mass index
CVD: Cardiovascular diseases
FINS: Fasting insulin
GDM: Gestational diabetes mellitus
HDL: High-density lipoprotein
HOMA-IR: The homeostatic model assessment of insulin resistance
lbHDL_2b_-P: Large buoyant high-density lipoprotein 2b particles
lbLDL-P: Large buoyant low-density lipoprotein particles
LDL: Low-density lipoprotein
OGTT: Oral glucose tolerance test
RLP: Remnant lipoprotein particles
sdLDL-P: Small dense low-density lipoprotein particles
VLDL: Very-low-density lipoprotein
